# Diagnostic differentiation of Zika and dengue virus exposure by analyzing T cell receptor sequences from peripheral blood of infected HLA-A2 transgenic mice

**DOI:** 10.1371/journal.pntd.0008896

**Published:** 2020-12-03

**Authors:** Mariah Hassert, Kyle J. Wolf, Ahmad Rajeh, Courtney Schiebout, Stella G. Hoft, Tae-Hyuk Ahn, Richard J. DiPaolo, James D. Brien, Amelia K. Pinto

**Affiliations:** 1 Department of Molecular Microbiology and Immunology, Saint Louis University- School of Medicine, Saint Louis, Missouri, United States of America; 2 Program in Bioinformatics and Computational Biology, Saint Louis University, Saint Louis, Missouri, United States of America; 3 Department of Computer Science, Saint Louis University, Saint Louis, Missouri, United States of America; University of Glasgow, UNITED KINGDOM

## Abstract

Zika virus (ZIKV) is a significant global health threat due to its potential for rapid emergence and association with severe congenital malformations during infection in pregnancy. Despite the urgent need, accurate diagnosis of ZIKV infection is still a major hurdle that must be overcome. Contributing to the inaccuracy of most serologically-based diagnostic assays for ZIKV, is the substantial geographic and antigenic overlap with other flaviviruses, including the four serotypes of dengue virus (DENV). Within this study, we have utilized a novel T cell receptor (TCR) sequencing platform to distinguish between ZIKV and DENV infections. Using high-throughput TCR sequencing of lymphocytes isolated from DENV and ZIKV infected mice, we were able to develop an algorithm which could identify virus-associated TCR sequences uniquely associated with either a prior ZIKV or DENV infection in mice. Using this algorithm, we were then able to separate mice that had been exposed to ZIKV or DENV infection with 97% accuracy. Overall this study serves as a proof-of-principle that T cell receptor sequencing can be used as a diagnostic tool capable of distinguishing between closely related viruses. Our results demonstrate the potential for this innovative platform to be used to accurately diagnose Zika virus infection and potentially the next emerging pathogen(s).

## Author summary

Diagnostic differentiation between dengue virus and Zika virus infections is a challenge due to serological cross-reactivity. In this study, we used a novel T cell receptor sequencing platform to identify T cell receptor sequences significantly associated with either dengue or Zika virus infection in HLA-A2 transgenic mice. These libraries were used to computationally train diagnostic classifiers which were capable of distinguishing between dengue and Zika virus in independent cohorts of infected mice.

## Introduction

Zika virus (ZIKV) is a member of the *Flaviviridae* family which includes West Nile (WNV), yellow fever (YFV), and the four serotypes of dengue (DENV1-4). Due to sharing a common vector, increased globalization, and climate change, many flaviviruses now co-circulate in the same geographic regions, resulting in far more than 50% of the world’s population being at risk of flavivirus exposure [1]. Moreover, because co-circulating flaviviruses are genetically and structurally very similar [2–9] antibodies generated in response to individual flaviviruses are often cross-reactive. These confounding factors, have resulted in serologically-based diagnostic assays that poorly differentiate between infections with the different flaviviruses [10,11]. Studies have reported that the false positive rate for ZIKV diagnosis, is as high as 40 percent when using ELISA based serological assays in flavivirus endemic areas [12].

Our inability to accurately distinguish flavivirus infections has resulted in a clouded picture of the current flavivirus epidemics, impacting both our preparedness and resource allocation. Following the introduction of ZIKV into the Western Hemisphere, studies began to show that the majority of adults exposed to ZIKV have few if any symptoms [13], however ZIKV could persist for more than six months in the semen of an infected individual [14] and be sexually transmitted as well as transmitted by mosquitos [15]. With the introduction of ZIKV into a naïve adult population it also became clear that ZIKV infection during pregnancy was associated with a significant number of infants born with congenital malformations termed “Congenital Zika Syndrome” (CZS), which manifest in various degrees of severity and have lifelong impacts on neurological and physical development [16]. Additionally, for adults, ZIKV infection is associated with Guillian-Barré Syndrome (GBS), encephalomyelitis, and seizures [17,18]. Notably, the risk associated with developing GBS following ZIKV exposure varies widely, as studies report that between 0.1 and 5 percent of ZIKV infections are associated with GBS [11,19,20]. One factor which has likely played a role in this variation is the ability to distinguish ZIKV exposure from other flaviviruses after the acute viremia has subsided. A diagnostic tool which can accurately distinguish ZIKV from related flaviviruses would inform surveillance and public health both within endemic regions and unaffected areas where the virus might spread.

The ability of naïve T cells to respond to any antigen is a necessity of the host defense system. T cells recognize immunogenic peptide antigens bound to MHC through binding of the T cell receptor (TCR) to the peptide-MHC complex (pMHC) [21]. The TCR is a heterodimeric protein comprised of TCRα and TCRβ chains. Individual TCR chains are generated through quasi-random genetic recombination from the germ-line loci of the variable (V), diversity (D), and joining (J) gene segments. Ultimately, the genetic rearrangement events result in an extremely high degree of diversity, particularly in the complementarity determining 3 region (CDR3) of the TCR that is comparable to the diversity found within the immunoglobulin (Ig) repertoire [22–24]. During a primary infection, recognition of a p-MHC complex by a TCR results in activation and expansion of a T cell with that given specificity [25,26]. Following pathogen clearance, a subset of responding T cells will be retained in a long-lived memory population of T cells that are present at a higher frequency than circulating naïve T cells, resulting in enrichment of those specific TCRs within peripheral blood. As T cells have a fixed TCR sequence, and do not undergo affinity maturation like antibody molecules (i.e. *the sequence of the TCR* stays consistent over time). Therefore, the enrichment of these unique DNA rearrangements can act as a stable biomarker that can be used to catalogue an individual’s immunological history of pathogen exposures [27,28].

Although many pathogen specific T cells are unique to an individual (private TCRs), a subset of identical antigen-specific TCRs can be found among multiple individuals (termed, “public” TCRs) [29–31]. The advancement in next-generation sequencing is allowing researchers to analyze the TCR repertoires with unprecedented depth and sensitivity, from very limited volumes of blood [27,29,32,33]. Therefore, while ZIKV and DENV antibody responses are largely cross-reactive and are indistinguishable by ELISA (S1 Fig), we hypothesize that we will be able to apply a TCRβ based sequencing technique and our optimized computational platform [34] to distinguish between ZIKV and DENV exposure. In support of this hypothesis, we previously demonstrated the use of TCRβ-specific high-throughput sequencing to survey the TCR sequences present in a peripheral blood and computationally identify public poxvirus-specific TCRβ sequences [34]. Additionally, we have previously shown that ZIKV infection of H2^b^-restricted B6 mice generates a robust CD4+ and CD8+ T cell response, which is preserved in memory and important for protection from a lethal challenge [35,36]. In a similar H2^b^-restricted model system for DENV2 infection, DENV-specific T cells have also been identified and shown to be preserved into memory [37,38].

To test our hypothesis, and determine if we could use virus specific TCR sequences to distinguish between DENV and ZIKV infections, we performed high-throughput TCRβ sequencing from circulating T cells isolated from peripheral blood of HLA-A2 transgenic mice prior to and after exposure to either ZIKV (strain PRVABC59) [39] or DENV2 (strain D2S20) [40]. From the sequence data we were able to create enriched pathogen specific public TCR libraries of ZIKV-associated TCRβ sequences (ZATS) and DENV2-associated TCRβ sequences (DATS). These libraries were used to train diagnostic classifiers, by calculating the ratio ZATS or DATS relative to the total number of unique TCRβ clonotypes. We then used the diagnostic classifier to identify ZIKV or DENV2 exposure within the training data set, as well as in independent cohorts of mice, infected with ZIKV, DENV2 or an unrelated virus (ACAM2000 smallpox vaccine). Within the independent data set, the diagnostic classifiers correctly diagnosed ZIKV or DENV2 infection in >97% of the samples. Overall, these data demonstrate that the virus-specific TCR repertoires generated in response to ZIKV or DENV2 infection include non-overlapping enriched TCRs in HLA-A2 transgenic mice that can be used to distinguish infection between the two viruses.

## Methods

### Ethics statement

All animal studies were conducted in accordance with the Guide for Care and Use of Laboratory Animals of the National Institutes of Health and approved by the Saint Louis University Animal Care and Use Committee (IACUC protocol # 2667).

### Lead contact

Further information and requests for resources and reagents should be directed to and will be fulfilled by the Lead Contact, Dr. Amelia Pinto (amelia.pinto@health.slu.edu).

### Mice

HLA-A2.1 AAD C57BL/6 mice were purchased from Jackson Laboratories and were housed and bred under specific pathogen free conditions at Saint Louis University. These mice express a transgenic HLA-A2.1 chimeric molecule containing human β-2 microblobulin and HLA-A2.1 α1 and α2 domains with a mouse α3 and transmembrane domain. The study was designed in the hopes of identifying HLA-A2 restricted T cell epitopes using a full length ZIKV peptide library, as previously described [35,36]. However, in our epitope mapping efforts, we were only able to identify T cell responses to previously published H2^b^ restricted epitopes [35,36]. Both male and female mice entered the study at 6–8 weeks of age, weighing between 18–23 and 16–21 grams respectively. Mice were housed in groups of 3–5 mice per cage. All animal work was conducted in accordance with the Guide for Care and Use of Laboratory Animals of the National Institutes of Health with approval from the Saint Louis University Institutional Animal Care and Use Committee.

### Viruses

ZIKV, strain PRVABC59 (GenBank accession KU501215.1) was obtained from BEI (Catalog number: NR-50240) and passaged in Vero-WHO cells (African green monkey kidney epithelial cells), which were purchased from American Type Culture Collection (ATCC CCL-81). ZIKV stocks were grown to a titer of 10^7^ focus forming units (FFU) per ml in DMEM before being harvested. Media supernatant was clarified of cells by centrifugation, aliquoted, and frozen at -80°C. The DENV2 used in this study (strain D2S20) (GenBank accession HQ891024), is a mouse adapted strain of dengue serotype 2 and was a kind gift from Dr. Michael Diamond [41]. The virus was grown in C6/36 *Aedes albopictus* cells (ATCC CRL-1660) in DMEM. At the time of harvest, media supernatant was clarified of cells by centrifugation. The virus was concentrated to a titer of 10^7^ FFU per ml by ultracentrifugation at 30,000 RPM in an SW-32 rotor for 3 hours over a 25% glycerol cushion before being aliquoted and frozen at -80°C [42].

### Infection and blood collection

At 6–8 weeks of age, HLA-A2 mice were anesthetized by intraperitoneal injection (IP) with a ketamine/xylazine cocktail and infected with 10^6^ FFU of either ZIKV or DENV2 by combination IP and subcutaneous footpad (SC) injection. For ZIKV and DENV2 to replicate to sufficient titers to cause disease in mice requires the inhibition of the type 1 interferon signaling cascade [43]. However, it has previously been demonstrated that C57BL/6 mice infected with ZIKV or DENV2 have detectable levels of viral RNA, sufficient for driving robust T cell responses that are detectable out to 30 days post infection [35,36,44]. We were able to confirm that indeed infection of HLA-A2 mice with ZIKV, generated a robust CD8+ and CD4+ T cell response. Approximately equal ratios of male and female mice were used in each infection condition. Blood samples were collected via submandibular bleed 1 week prior to infection (un-infected) and 8, 18, and approximately 127 days after infection.

### DIII ZIKV and DENV ELISA

Serum was collected from HLA-A2 mice prior to infection and at day 18 post infection with ZIKV or DENV2 and tested for reactivity to domain III of ZIKV or DENV envelope protein. 96-well maxSorp plates (ThermoFisher 12-565-136) were coated with 5 μg per ml purified ZIKV domain III in carbonate buffer (0.1M Na_2_CO_3,_ 0.1M NaHCO_3,_ pH 9.3) and incubated overnight at 4°C. Wells were washed and blocked with 200 μl of blocking buffer (PBS + 5% BSA + 0.1% Tween) for 1 hour at 37°C. Plates were washed with PBS and 50 μl of diluted serum (1:20 in blocking buffer) was added to each well and incubated for 1h at 37°C. Plates were washed with PBS and 50 μl of goat anti-mouse HRP-conjugated antibody (Sigma A8924, diluted 1:5000 in blocking buffer) was added and allowed to incubate for 1 hour at room temperature. Plates were washed with PBS and 100 μl of True Blue™ peroxidase substrate (SeraCare 71-00-65) was added to each well and incubated for 15 minutes, in the dark, at room temperature. 100 μl of 1N hydrochloric acid was added to each well and an OD measurement was recorded at 450 nm.

### Sample preparation and DNA sequencing

Genomic DNA was extracted from whole blood or cells using the QIAGEN DNeasy Blood and Tissue Kit (QIAGEN 69506) according to the manufacturer’s instructions. Genomic V and J segments were amplified in an unbiased manner using a set of multiplexed primers targeting all V and J gene segments as previously described [45]. TCRβ CDR3 regions were further amplified and sequenced using ImmunoSEQ^TM^ (Adaptive Biotechnologies). Synthetic templates mimicking natural V(D)J rearrangements were used to measure and correct for potential amplification bias at this stage [32,45]. CDR3 segments were annotated according to the International ImMunoGeneTics (IMGT) collaboration [46], identifying V, D, and J genes contributing to each rearrangement.

### Peptide stimulation of splenocytes

At 18 days post-primary infection, spleens from ZIKV infected HLA-A2 male and female mice were ground over a 70μm cell strainer and washed with RPMI (Sigma Aldrich R8758) supplemented with 10% fetal bovine serum (Sigma Aldrich F0926), 1% HEPES (Sigma Aldrich H3537), and 1X beta-mercaptoethanol (Gibco 21985023). Approximately 10^6^ cells were stimulated in the presence of 10 μg per ml of brefeldin A for 6 hours in each well of a 96 well round bottom plate with 10 μg of pooled 9-mer or 15-mer peptides (21^st^ Century Biochemicals). The peptides used were previously published as ZIKV MHC class I and II H2^b^ restricted epitopes respectively [35,36].

### Flow cytometric sorting of T cells

Following peptide stimulation in a 96 well plate, cells were washed with PBS and wells receiving identical treatments of the same mouse’s cells were pooled. Cells were stained overnight at 4°C for the following surface markers in PBS: CD19-AF488 (clone 1D3), CD4-APC-Cy7 (clone RM4-5), and CD8-PerCP-Cy5.5 (clone 53–6.7). Cells were washed with PBS and fixed with 2% paraformaldehyde (Fisher 15713-S) for 5 minutes at 4°C. Cells were washed and permeablized with 0.5% saponin (Sigma 47036-250G) prior to intracellular cytokine staining. Cells were stained for 1 hour at 4°C for the following intracellular cytokines: IFNγ-APC (clone B27) and TNFα-PE (clone Mab11). Cells were gated and sorted into two populations in the following manner: Lymphocytes ➔ CD19+ ➔ CD4+/CD8- ➔ IFNγ+ or Lymphocytes ➔ CD19+ ➔ CD4-/CD8+ ➔ IFNγ+ (S2 Fig). Cells were sorted using a BDFACS Aria-Fusion cell sorter and collected into PBS.

### Quantification and statistical analysis

The identification of public TCRβ sequences associated with ZIKV or DENV2 infection in HLA-A2 mice and the diagnostic classifiers used to discriminate between DENV2 and ZIKV infection in these animals was completed using the identical platform as previously described [34]. A methods manuscript with the specific command line used in these studies is currently in preparation (Rajeh et al.).

*ZATS and DATS library development*: The development of the DATS and ZATS libraries were completed as previously described by our groups [34]. Briefly, the sequencing output was aligned using the software ImmunoSEQ^TM^. The sequencing data from day 8 and day 18 post ZIKV or DENV2 infection were collectively used to identify public TCR sequences. The public TCR sequences found in ZIKV or DENV2 infected mice were then compared to the aligned sequences containing the TCRβ sequences from the naïve mice. We performed an association analysis to identify any TCRβ sequences that had significantly increased in incidence in the ZIKV or DENV2 infected mice compared to the naïve mice by a one-tailed Fisher’s Exact test. We applied a heuristic test to determine the optimal p-value threshold based upon the coverage provided by the library for both infected (C_v_) and naïve samples (C_n_) [34]. “Coverage” in this case, is defined as the summation of the number of samples containing TCRβ sequence (x_i_ or y_i_, infected or naïve, respectively) divided by the total number of sequences in the training data (n_v_ or n_n_, infected or naïve, respectively).

$$C_{v}=\frac{\sum_{i=1}^{I}x_{i}}{n_{v}}\;C_{n}=\frac{\sum_{i=1}^{I}y_{i}}{n_{n}}$$

The C_v_ to C_n_ ratio is determined for each unique p value and is applied to a one-tailed Fisher’s Exact test against the total number of sequences in the prospective library to determine if there will be sufficient coverage to distinguish infected from naïve samples (p<0.05).

*Classification of ZIKV or DENV2 exposed samples*: To distinguish between ZIKV or DENV2 exposed versus unexposed, the proportion of ZATS or DATS identified in an individual sample were compared against the normal distribution of the infected and naïve training data set as described in Wolf et al., 2018 [34]. The normal distributions were calculated based on a function of the difference between a single sample value (x) and the mean of a set of data (μ) over the standard deviation of that set of data (σ), utilizing a correction constant to remove negative selection bias of the naïve training sample data. In this case, a larger value has a greater association with the training group.

$$f(x|{\mu ,\;\sigma }^{2})=\left(\frac{1}{\sqrt{2{\pi \sigma }^{2}}}\right)e^{-\frac{{\left(x-\mu \right)}^{2}}{2\sigma^{2}}}$$

*AUROC curve generation*: AUROC curves were generated using GraphPad Prism by plotting the sensitivity (true positive) by the false discovery rate (FDR), representing the overall accuracy.

## Results

### Building of ZIKV and DENV-associated TCR libraries

**Fig 1:**
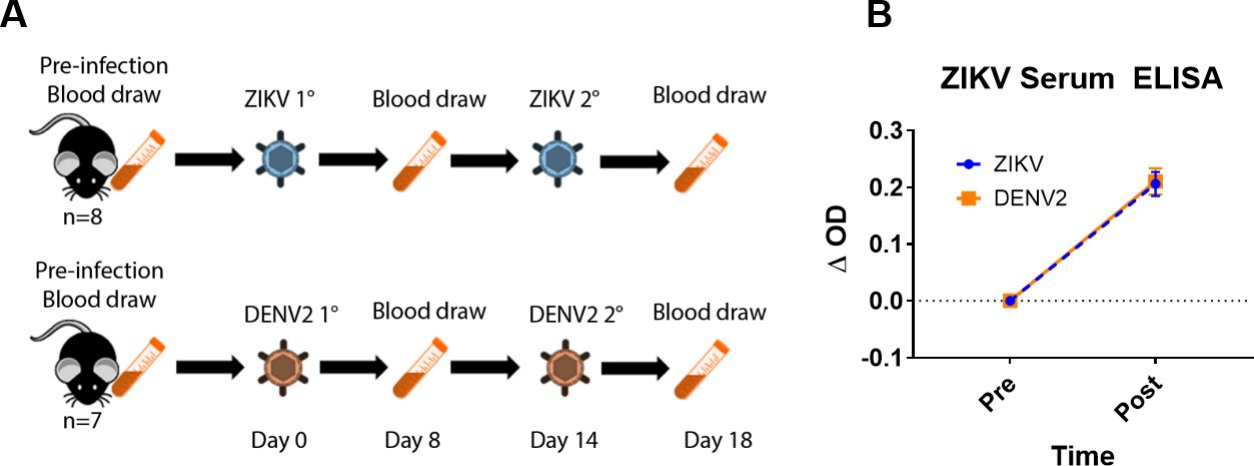
Experimental design. (**A**) 8 week-old HLA-A2 transgenic mice were infected with either ZIKV (n = 8) or DENV2 (n = 7). The animals were boosted with the homologous virus 14 days post primary infection to further boost the frequency of antigen-specific T cells. Blood was collected prior to infection, at day 8, and day 18 post infection and prepared for TCR sequencing. (**B**) ZIKV DIII-specific ELISA. Serum was collected from mice prior to DENV2 or ZIKV exposure and after DENV2 or ZIKV exposure. A ZIKV DIII ELISA was done to determine if an antibody based assay could be used to distinguish between ZIKV and DENV2 infection.

As a proof-of-principle, we set out to determine if TCRβ sequencing could be used in a diagnostic assay capable of discriminating between infection with DENV and ZIKV, two serologically cross-reactive flaviviruses (S1 Fig). 6–8 week old HLA-A2 transgenic mice were infected with either ZIKV (strain PRVABC59) (n = 8) or DENV2 (strain D2S20) (n = 7) to elicit a robust T cell response. Fourteen days following primary infection, the mice were boosted with the homologous virus to further expand the frequency of antigen-specific cells. We sampled the TCR repertoires of these mice by collecting blood from each animal on day 0 (prior to infection/exposure), day 8, and day 18 post infection (Fig 1A). High-throughput sequencing and bioinformatic analyses were performed to identify the TCRβ clonotypes in each sample, as previously described [34]. Within this study, we defined a “unique TCRβ clonotype” as a unique combination of V gene, CDR3 amino acid sequence, and J gene (Table 1). To confirm infection and serological antigenic overlap, serum was collected at day 0 and day 18 from each mouse and used to measure ZIKV reactive antibodies using a ZIKV envelope domain III (DIII) ELISA. There were no measurable differences in the quantity of ZIKV DIII reactive antibody between the sera of ZIKV and DENV2 infected mice (Fig 1B). These results demonstrate that in our models, as has been seen in human diagnostic assays, antibody cross-reactivity between related flaviviruses does occur and convolutes serologically-based diagnostic assays.

**Table 1:** TCRβ Sequence rearrangements and clonotypes in infected and uninfected mice.

Treatment Group	# Samples	# Unique Clonotypes	Total TCR Rearrangements
Naïve	n = 15	4.25x10^5^	8.78x10^5^
ZIKV	n = 13	4.01x10^4^	8.02x10^4^
DENV2	n = 11	3.89x10^4^	7.47x10^4^

Consolidated data referencing the total number of mice, unique TCRβ sequences (clonotypes), and total number of rearranged TCRβ genes sequenced in the Naïve, ZIKV infected, and DENV2 infected groups.

**Fig 2:**
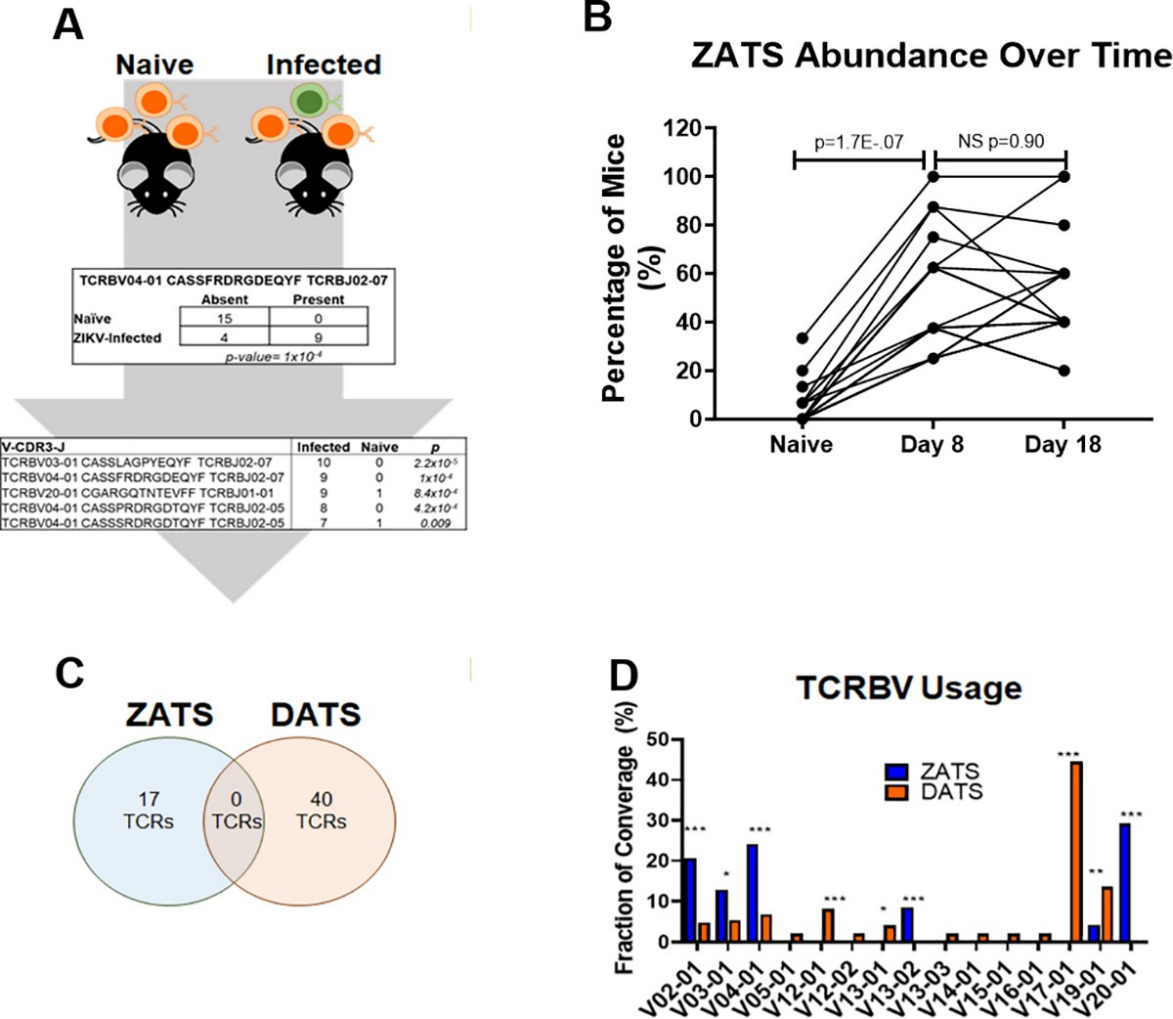
Generation of ZIKV- and DENV2-associated TCRβ sequencing libraries. (**A**) Genomic DNA was extracted from whole blood and sequenced via Adaptive Biotechnologies. TCRβ repertoires from infected mice were compared to those of naïve mice and an association analysis was used to identify specific TCRβ that had significantly increased following infection. (**B**) To determine if the overall abundance of ZAT sequences changed in the ZIKV infected cohorts of mice between day 0, primary infection, and boost, the percentage of mice that each ZAT was found in was plotted at each time point. From day 0 to day 8, the percentage of mice that each ZAT was found in increased significantly. However, from primary to secondary infection (day 8 to day 18), there was no overall increase in the percentage of mice containing these ZATS. Statistical significance was determined by a paired t test. (**C**) Venn-diagram displaying the overlap of TCRβ clonotypes between the ZATS and DATS libraries. By comparing the sequences in the ZATS and DATS libraries, we found that TCR repertoires generated in response to ZIKV or DENV2 infection include non-overlapping enriched TCRs in HLA-A2 transgenic mice. (**D**) TCRVβ usage as a proportion of all ZATS or DATS identified in ZIKV or DENV2 infected mice. There was significant enrichment of Vβ gene usage towards TCRBV02-01, V04-01 and V20-01 in the ZATS library compared to increased TCRBV17-01 and V19-01 gene usage in the DATS library. Statistical significance was calculated using a Fisher’s Exact test (* p<0.05, ** p<0.01, *** p<0.001).

The sequences that were obtained from these mice were analyzed to computationally identify specific TCRβ clonotypes that were associated with ZIKV or DENV2 infection by enumerating the number of samples each TCRβ was present in, and comparing those to TCRβ clonotypes present prior to challenge (naïve). A Fisher’s Exact Test was used to identify specific TCR sequences that were associated with ZIKV or DENV2 infection (Fig 2A). In the case of ZIKV infection, the analysis generated a library of 17 ZIKV-associated TCR sequences (ZATS) (Table 2). TCR repertoires from mice infected with DENV2 were analyzed using the same approach and a library of DENV2-associated TCRs (DATS) consisting of 40 TCRβ sequences was constructed (Table 2). Between the primary and secondary infection, we found that the relative abundance of these virus associated TCRs did not significantly change (Fig 2B), and therefore, for increased statistical power, sequences from day 8 and 18 were analyzed together. When we compared the ZATS and DATS libraries, we found that there was no overlap of the virus-associated TCRβ sequences (Fig 2C). We noted a significant skewing of Vβ gene usage towards TCRBV02-01, V04-01 and V20-01 in the ZATS library compared to increased TCRBV17-01 and V19-01 gene usage in the DATS library (Fig 2D). Collectively, these results demonstrate that a subset of the TCR repertoires for the DENV2 and ZIKV mice are unique to the infecting virus.

**Table 2:** ZIKV or DENV2 associated TCRβ sequence libraries.

	V-CDR3-J	# Exposed	#Naive	p-value
ZIKV Associated TCRS	TCRBV03-01 CASSLAGPYEQYF TCRBJ02-07	10 of 13	0 of 15	*2*.*18E-05*
	TCRBV04-01 CASSFRDRGDEQYF TCRBJ02-07	9 of 13	0 of 15	*1*.*04E-04*
	TCRBV20-01 CGARGQANTEVFF TCRBJ01-01	13 of 13	5 of 15	*2*.*29E-04*
	TCRBV04-01 CASSPRDRGDTQYF TCRBJ02-05	8 of 13	0 of 15	*4*.*14E-04*
	TCRBV20-01 CGARGQTNTEVFF TCRBJ01-01	9 of 13	1 of 15	*8*.*39E-04*
	TCRBV02-01 CASSQGTGGNYAEQFF TCRBJ02-01	11 of 13	3 of 15	*9*.*19E-04*
	TCRBV20-01 CGARGQSNTEVFF TCRBJ01-01	7 of 13	0 of 15	*1*.*45E-03*
	TCRBV04-01 CASSSRDRGDTQYF TCRBJ02-05	7 of 13	1 of 15	*8*.*70E-03*
	TCRBV19-01 CASSIGDRGREQYF TCRBJ02-07	5 of 13	0 of 15	*1*.*31E-02*
	TCRBV20-01 CGASRDRGQAPLF TCRBJ01-05	5 of 13	0 of 15	*1*.*31E-02*
	TCRBV02-01 CASSRGTGGNYAEQFF TCRBJ02-01	4 of 13	0 of 15	*3*.*49E-02*
	TCRBV04-01 CASSKRDRGDTQYF TCRBJ02-05	4 of 13	0 of 15	*3*.*49E-02*
	TCRBV02-01 CASSHGTGGNYAEQFF TCRBJ02-01	4 of 13	0 of 15	*3*.*49E-02*
	TCRBV13-02 CASGPQGSQNTLYF TCRBJ02-04	4 of 13	0 of 15	*3*.*49E-02*
	TCRBV02-01 CASSQGGGSSYEQYF TCRBJ02-07	5 of 13	1 of 15	*5*.*58E-02*
	TCRBV03-01 CASSLAGSYEQYF TCRBJ02-07	5 of 13	1 of 15	*5*.*58E-02*
	TCRBV13-02 CASGETGNQDTQYF TCRBJ02-05	6 of 13	2 of 15	*6*.*67E-02*
	**V-CDR3-J**	**# Exposed**	**#Naive**	***p-value***
DENV2 Associated TCRs	TCRBV19-01 CASSIPAEVFF TCRBJ01-01	6 of 11	0 of 15	*2*.*01E-03*
	TCRBV12-01 CASSLGTGGANTGQLYF TCRBJ02-02	5 of 11	0 of 15	*7*.*02E-03*
	TCRBV02-01 CASSPTNSGNTLYF TCRBJ01-03	4 of 11	0 of 15	*2*.*21E-02*
	TCRBV03-01 CASSWDRSGNTLYF TCRBJ01-03	4 of 11	0 of 15	*2*.*21E-02*
	TCRBV17-01 CASSSGGDTEVFF TCRBJ01-01	4 of 11	0 of 15	*2*.*21E-02*
	TCRBV04-01 CASSSPFEQYF TCRBJ02-07	3 of 11	0 of 15	*6*.*35E-02*
	TCRBV17-01 CASRGAYEQYF TCRBJ02-07	3 of 11	0 of 15	*6*.*35E-02*
	TCRBV17-01 CASSRGPGDAEQFF TCRBJ02-01	3 of 11	0 of 15	*6*.*35E-02*
	TCRBV17-01 CASSTGTGVEQYF TCRBJ02-07	3 of 11	0 of 15	*6*.*35E-02*
	TCRBV13-03 CASWGGAEQFF TCRBJ02-01	3 of 11	0 of 15	*6*.*35E-02*
	TCRBV12-01 CASSLGTGGGNTGQLYF TCRBJ02-02	3 of 11	0 of 15	*6*.*35E-02*
	TCRBV17-01 CASSRGTGVSDYTF TCRBJ01-02	3 of 11	0 of 15	*6*.*35E-02*
	TCRBV17-01 CASSSGTGVEQYF TCRBJ02-07	3 of 11	0 of 15	*6*.*35E-02*
	TCRBV17-01 CASRDIYEQYF TCRBJ02-07	3 of 11	0 of 15	*6*.*35E-02*
	TCRBV17-01 CATRGAYEQYF TCRBJ02-07	3 of 11	0 of 15	*6*.*35E-02*
	TCRBV17-01 CASSPGTGWEQYF TCRBJ02-07	3 of 11	0 of 15	*6*.*35E-02*
	TCRBV17-01 CASRSSYEQYF TCRBJ02-07	3 of 11	0 of 15	*6*.*35E-02*
	TCRBV13-01 CASSDATDYEQYF TCRBJ02-07	3 of 11	0 of 15	*6*.*35E-02*
	TCRBV17-01 CASSAGTGGAEQFF TCRBJ02-01	3 of 11	0 of 15	*6*.*35E-02*
	TCRBV19-01 CASSIGTYYAEQFF TCRBJ02-01	3 of 11	0 of 15	*6*.*35E-02*
	TCRBV17-01 CASSREYAEQFF TCRBJ02-01	3 of 11	0 of 15	*6*.*35E-02*
	TCRBV17-01 CASRNSYEQYF TCRBJ02-07	3 of 11	0 of 15	*6*.*35E-02*
	TCRBV16-01 CASSDRTGAYEQYF TCRBJ02-07	3 of 11	0 of 15	*6*.*35E-02*
	TCRBV12-02 CASSPDWGDNYAEQFF TCRBJ02-01	3 of 11	0 of 15	*6*.*35E-02*
	TCRBV19-01 CASSPGTEYEQYF TCRBJ02-07	3 of 11	0 of 15	*6*.*35E-02*
	TCRBV15-01 CASSLGGSSSAETLYF TCRBJ02-03	3 of 11	0 of 15	*6*.*35E-02*
	TCRBV19-01 CASSPGGDAEQFF TCRBJ02-01	3 of 11	0 of 15	*6*.*35E-02*
	TCRBV14-01 CASSQGSQNTLYF TCRBJ02-04	3 of 11	0 of 15	*6*.*35E-02*
	TCRBV04-01 CASSTTEVFF TCRBJ01-01	3 of 11	0 of 15	*6*.*35E-02*
	TCRBV13-01 CASSGRDRGNERLFF TCRBJ01-04	3 of 11	0 of 15	*6*.*35E-02*
	TCRBV05-01 CASSQGGWAETLYF TCRBJ02-03	3 of 11	0 of 15	*6*.*35E-02*
	TCRBV02-01 CASSQDRGANQDTQYF TCRBJ02-05	3 of 11	0 of 15	*6*.*35E-02*
	TCRBV19-01 CASSIPTEVFF TCRBJ01-01	5 of 11	1 of 15	*3*.*21E-02*
	TCRBV12-01 CASSPGTGGANTGQLYF TCRBJ02-02	4 of 11	1 of 15	*8*.*23E-02*
	TCRBV04-01 CASSSTEVFF TCRBJ01-01	4 of 11	1 of 15	*8*.*23E-02*
	TCRBV03-01 CASSPGLDYAEQFF TCRBJ02-01	4 of 11	1 of 15	*8*.*23E-02*
	TCRBV17-01 CASSPGTGDTEVFF TCRBJ01-01	4 of 11	1 of 15	*8*.*23E-02*
	TCRBV17-01 CASSRSAETLYF TCRBJ02-03	4 of 11	1 of 15	*8*.*23E-02*
	TCRBV17-01 CASSRAYEQYF TCRBJ02-07	8 of 11	2 of 15	*3*.*42E-03*
	TCRBV17-01 CASSRTGGYEQYF TCRBJ02-07	9 of 11	7 of 15	*7*.*76E-02*

**Fig 3:**
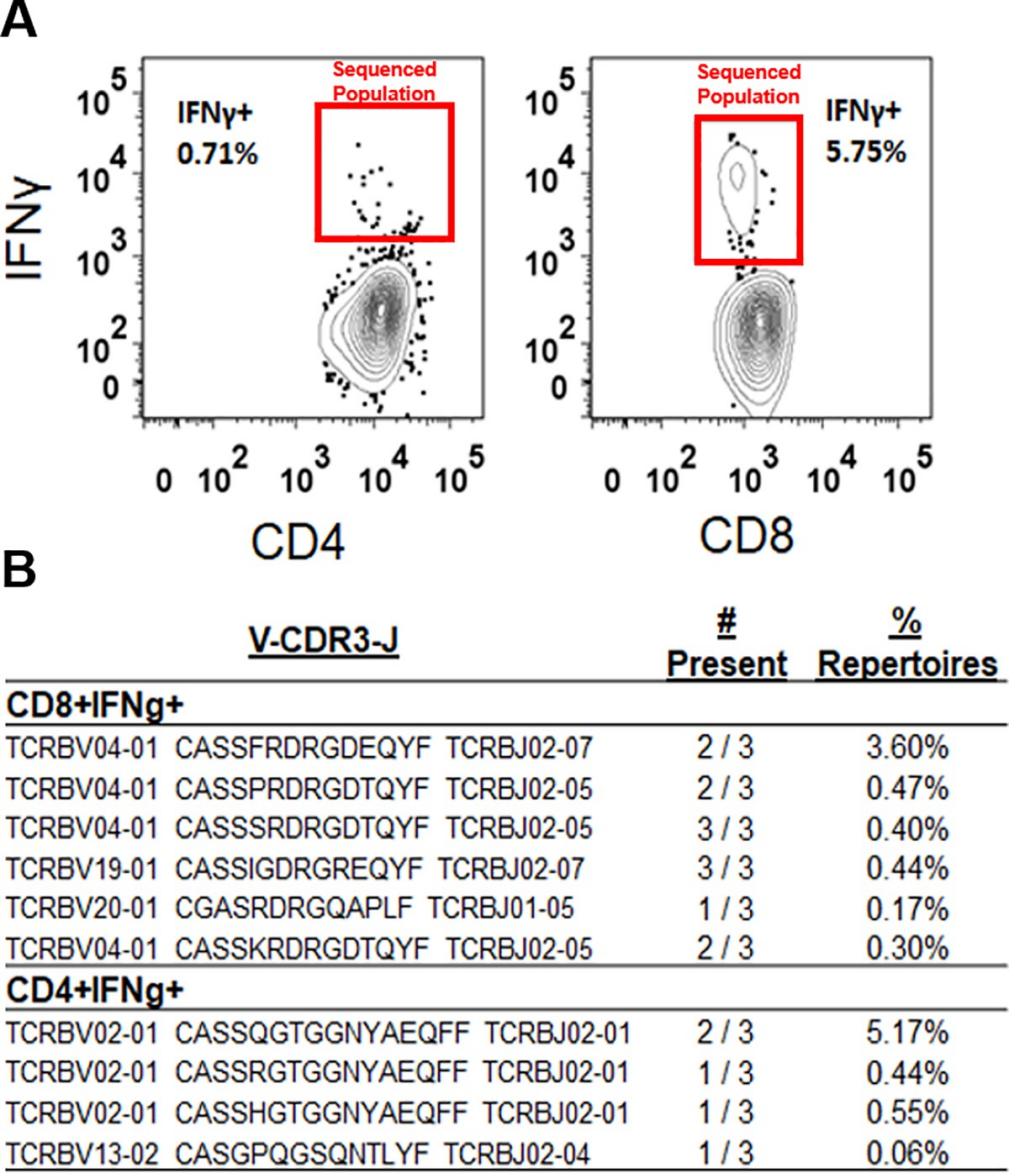
Enrichment of ZATS sequences in functional, ZIKV-specific T cells. (**A**) Representative flow plots of ZIKV-specific IFNγ responses after peptide stimulation of CD8+ (left) and CD4+ (right) T cells. Splenocytes from ZIKV infected mice were stimulated with published MHC-I and MHC-II restricted ZIKV specific peptides (Table 3) [35, 36]. Following stimulation, cells were stained and sorted based on IFNγ production in CD4+ or CD8+ T cells. (**B**) TCRβ sequences from the ZATS library that were identified in sorted ZIKV-specific CD4+ and CD8+ T cells. After sorting, genomic DNA was prepared from ZIKV-reactive CD4+ or CD8+ T cells and sequenced. 6 of the 17 ZATS were identified in the sorted ZIKV-reactive CD8+ T cell population and 4 of the 17 ZATS were identified in the sorted ZIKV-reactive CD4+ T cell population.

To further validate our method of library construction, we sought to determine if the 17 public TCRβ sequences composing the ZATS library, were particularly enriched in sorted populations of ZIKV-reactive T cells. To test this, we stimulated splenocytes from three ZIKV infected mice in the presence of brefeldin A, with pools of peptides that we had previously shown to be immunodominant MHC-I and MHC-II restricted ZIKV-specific epitopes [35,36] (Table 3). We found that using the pooled peptides epitopes approximately 0.7–1% of the CD4+ T splenocytes were reactive to the ZIKV CD4+ T cell pooled peptide epitopes and approximately 5–6% of the CD8+ T cells were reactive to the ZIKV CD8+ T cell pooled peptide epitopes (Fig 3A and S2 Fig). We then sorted CD4+IFNγ and CD8+IFNγ expressing T cells from each of the three mice to isolate these ZIKV-reactive T cell populations. Following DNA isolation, we sequenced the TCRs expressed by the sorted CD4+ and CD8+ T cells. From these enriched populations we compared the TCRβ clonotypes to those in the ZATS library generated from a separate cohort of infected mice. Six previously identified ZATS were present in the sorted ZIKV-reactive CD8+ T cells from at least one of mice and two ZATS were present in the sorted CD8+ T cells from all three mice. Notably, the six previously identified ZATS were present at high frequencies within this population in the individual mice (Fig 3B). Four of the 17 ZATS were also identified in the sorted ZIKV-reactive CD4+ T cells from at least one of mouse population, accounting for a relatively high frequency of the sequences within this population (Fig 3B). These data demonstrated that the computationally generated ZATS library contain sequences expressed by ZIKV-reactive T cells specific for known peptide epitopes.

**Table 3:** Peptide epitopes used for ex vivo restimulation.

Epitope	Amino Acid Sequence	MHC Restriction	Reference
E_294_	[H]IGVSNRDFV[OH]	D^b^	[35]
PrM_251_	[H]IFRNPGFALAAAAIA[OH]	I-A^b^	[36]
E_646_	[H]GRLITANPVITESTE[OH]	I-A^b^	[36]
NS_1811_	[H]TGVFVYNDVEAWRDR[OH]	I-A^b^	[36]
NS5_3211_	[H]KDTQEWKPSTGWDNW[OH]	I-A^b^	[36]

18 days post infection, splenocytes were harvested from ZIKV infected mice and stimulated with published ZIKV epitopes.

### Diagnostic discrimination of ZIKV and DENV infected mice

We have previously shown in the case of smallpox vaccinated mice (ACAM2000), that we can utilize the enrichment of virus-specific public TCR sequences to train a diagnostic classifier capable of distinguishing between naïve and vaccinated animals using a small sample of blood [34]. We hypothesized that because we were able to identify unique public ZIKV-associated TCR sequences and DENV-associated TCR sequences, that we would be able to train diagnostic classifiers capable of distinguishing between ZIKV and DENV2 infection. To this end, we developed diagnostic classifiers by calculating the ratio ZATS or DATS relative to the total number of unique TCRβ clonotypes present in each individual sample, as a means of evaluating clonal enrichment as previously described [34].

$$\frac{\#ZATS\;(or\;DATS)\;present}{\#Unique\;TCR\;Clonotypes}$$

**Fig 4:**
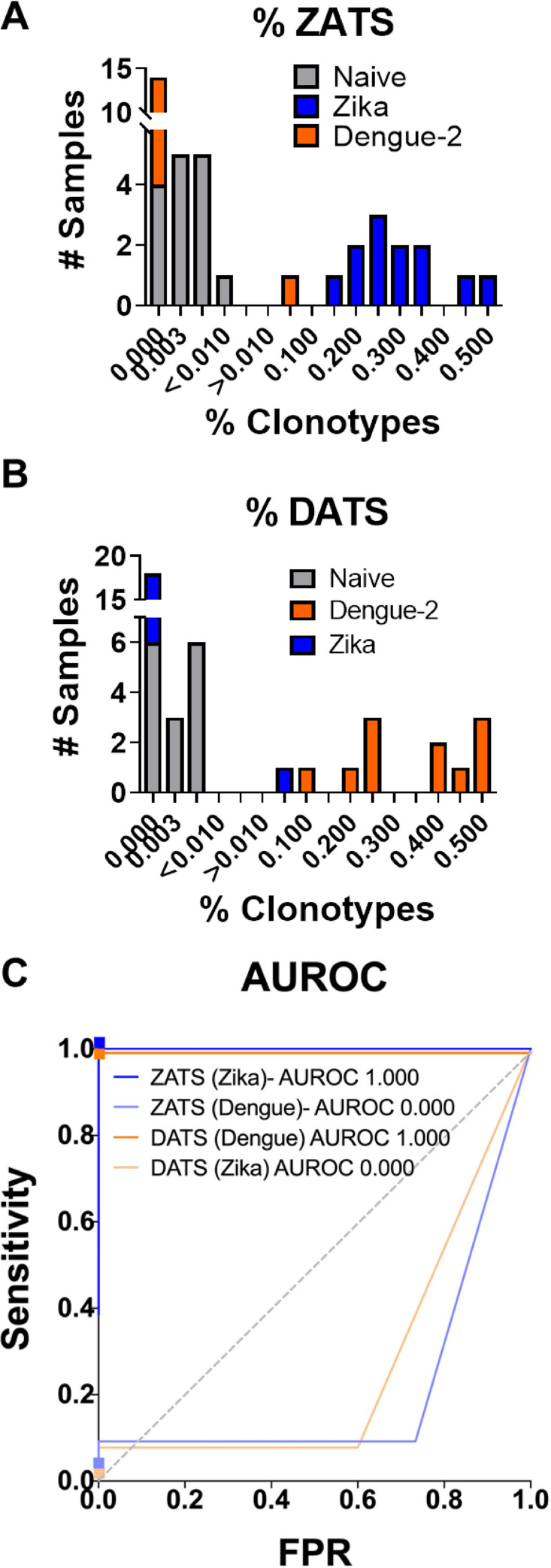
Diagnostic classification of ZIKV and DENV2 exposure within the training data set. (**A**) The ZATS ratio present in individual samples from naïve (gray), ZIKV infected (blue), or DENV2 infected (orange) mice.The ZATS ratio was determined by dividing the number of ZATS present by the total number of unique TCRβ clonotypes present in an individual sample. The ZATS library sequences were particularly enriched in ZIKV infected animals. (**B**) The DATS ratio present in individual samples from naïve (gray), ZIKV infected (blue), or DENV2 infected (orange) mice.The DATS ratio was determined by dividing the number of DATS present by the total number of unique TCRβ clonotypes present in an individual sample. (**C**) AUROC curve demonstrating the sensitivity of ZIKV and DENV2 diagnostic classifiers. The ZATS and DATS ratios were used to train diagnostic classifiers capable of discriminating between naïve, DENV2, and ZIKV infected mice within the training data set (see methods). 100% of ZIKV infected mice were correctly categorized as “ZIKV exposed” and no naïve or DENV2 infected mice were categorized as “ZIKV exposed.” Similarly, 100% of DENV2 infected mice were correctly categorized as “DENV exposed” and no naïve or ZIKV infected mice were categorized as “DENV exposed”.

A binary classifier was constructed to differentiate naïve and vaccinated samples on the basis of the normal distribution of percent ZATS or DATS as [28]. When evaluating the TCRβ repertoire of ZIKV infected mice, we noted a 95-fold increase in the ratio of ZATS (average 0.305%± 0.151%) relative to the TCRβ repertoire of naïve mice (average 0.003%± 0.002%), and a 205-fold increase in the ratio of ZATS relative to the TCRβ repertoire of DENV2 infected mice (average 0.001%±0.005%) (Fig 4A). This ZATS ratios in naïve and ZIKV-infected samples were used to train a statistical classifier, which was then used to diagnose the exposure status of the naïve, ZIKV infected, or DENV2 infected mice within the training data set (Fig 4C). 100% (13 of 13) of the ZIKV-infected mice were correctly diagnosed as “ZIKV exposed.” Importantly, 100% (15 of 15) of the naïve mice and 100% (11 of 11) of DENV2 infected mice were correctly diagnosed as ZIKV unexposed.

When evaluating the TCRβ repertoires of DENV2 infected mice, we noted a 147-fold increase in the ratio of DATS (average 0.367%± 0.210%) relative to the TCRβ repertoire of naïve mice (average 0.003%± 0.002%) and a 204-fold increase in the ratio of DATS relative to the TCRβ repertoire of ZIKV infected mice (average 0.002%±0.007%) (Fig 4B). Similarly, the DATS ratios in naïve and DENV2-infected samples were used to train a DENV2 diagnostic classifier (Fig 4C). 100% (11 of 11) of the DENV2 infected mice within the training data set were correctly diagnosed as DENV2 exposed, according to this classifier. 100% (15 of 15) of the naïve mice and 100% (13 of 13) of the ZIKV-infected mice were also correctly classified as “DENV2 unexposed.” These data show that the ZIKV and DENV2 diagnostic assays developed using the training ZATS and DATS libraries not only have the ability to accurately distinguish between exposed and naïve samples, but can distinguish between mice infected with DENV2 or ZIKV with a high degree of accuracy among the training data samples.

### Validation of diagnostic classifiers in an independent cohort of mice

**Fig 5:**
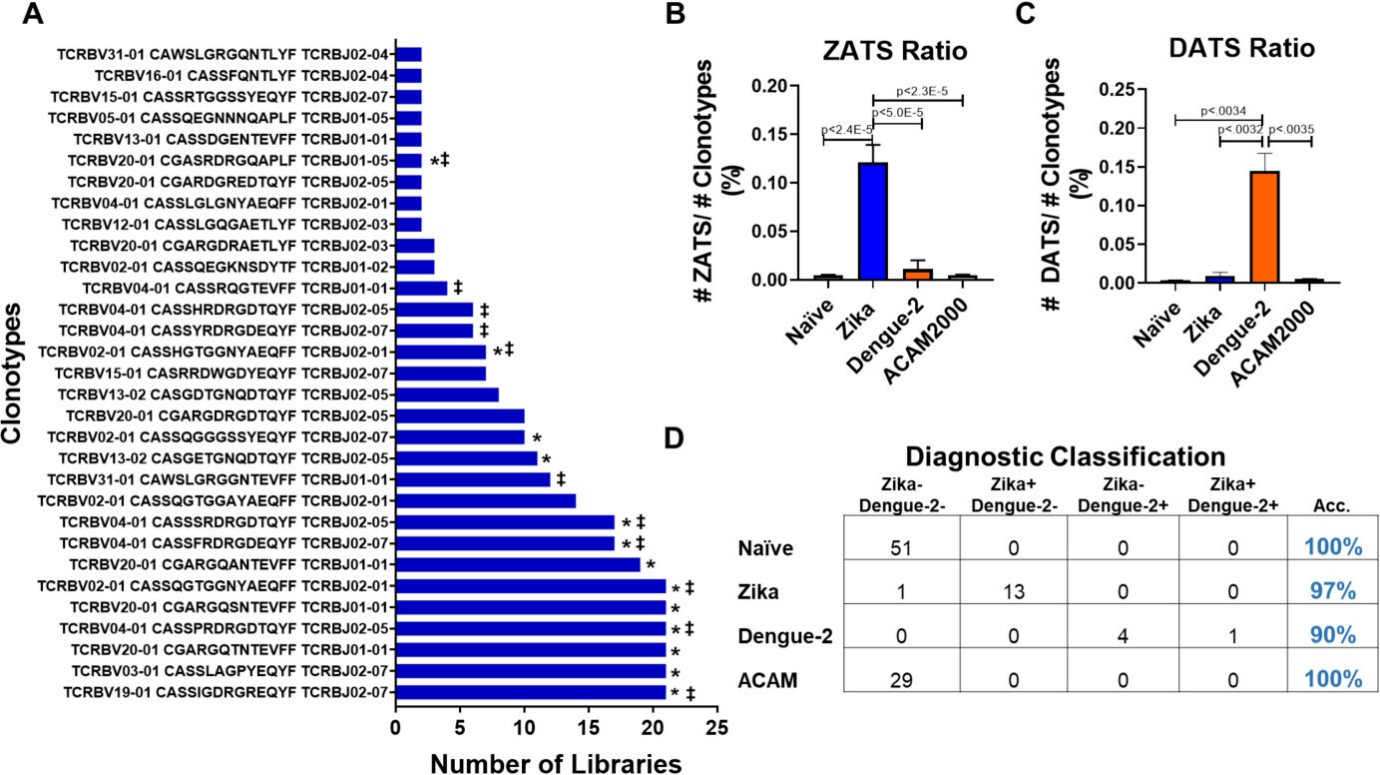
Validation of ZIKV and DENV2 diagnostic classifiers in an independent cohort of mice. (**A**) Permutation analysis of TCRβ sequencing data. Additional cohorts of mice were added to the training data set and permutation analysis was done 21 times to generate 21 ZATS libraries. The sequences found in the original ZATS library were subsequently identified within these libraries. (**B-C**) ZATS (**B**) and DATS (**C**) ratios present among independent cohorts of uninfected (gray), ZIKV infected (blue), DENV2 infected (orange), and ACAM2000 vaccinated (white) mice. Statistical significance was determined by 2-tailed unpaired t-test (**D**) Accuracy of diagnostic classifier when determining the infection state of independent cohorts of mice. TCRβ sequences were determined from peripheral blood lymphocytes of independent cohorts of uninfected mice or mice infected with ZIKV, DENV2, or ACAM2000. Mice in each infection condition were categorized as either ZIKV and DENV2 unexposed (ZIKV- DENV2-), ZIKV exposed and DENV2 unexposed (ZIKV+ DENV2-), ZIKV unexposed and DENV2 exposed (ZIKV- DENV2+), or exposed to both ZIKV and DENV2 (ZIKV + DENV2+).

To determine the reproducibility of our ZATS library, TCRβ sequencing data from multiple cohorts of mice pre- and post ZIKV exposure were incorporated into our training data set to perform permutation analysis. Briefly, pre- and post-infection samples were randomized, and a selection of samples pre- (n = 14–15) and post- (n = 13–14) infection were used to generate new ZATS TCR libraries; this randomization process was completed 21 times for 21 library permutations. From all 21 libraries, a total of 31 unique clonotypes were identified in at least 2 of the 21 libraries (Fig 5A). Of the 31 clonotypes, 13 clonotypes were found in present in the original ZATS library (denoted by a “*” in Fig 5A). Additionally, 11 listed clonotypes were identified in populations of purified ZIKV-specific CD8^+^IFNγ^+^ and CD4^+^IFNγ^+^ T cells (denoted by ‡)). These data support our hypothesis that public virus- specific TCRs can be consistently identified in multiple populations of mice and that the diagnostic platform can repeatedly identify the same virus-specific clonotypes given a randomized sampling of exposed samples.

While our diagnostic classifiers were accurate 100% of the time when discriminating between DENV2 and ZIKV infected animals within the training data set (Fig 4), it was still unclear the consistency at which these diagnostic classifiers could distinguish between the infections using sequencing data from independent cohorts of mice. To this end, we sequenced the TCR repertoires from independent cohorts of mice, before and after exposure to ZIKV, DENV2, or the live attenuated smallpox vaccine ACAM2000 (Table 4). The ratio of ZATS and DATS to the total number of clonotypes (indicating enrichment) was calculated in the TCR sequences from 51 naïve mice, 14 ZIKV infected mice, and 5 DENV2 infected mice. We found that within these independent cohorts of mice, that ZATS were significantly enriched in only the TCR repertoires of ZIKV infected mice (0.121%±0.068%) compared to naïve (0.005%± 0.005%), DENV2-infected (0.011%± 0.005%), or ACAM2000-vaccinated samples (0.005%± 0.005%) (Fig 5B). Similarly, the DATS were significantly enriched in only the DENV2 infected mice (0.145%± 0.051%) compared naïve (0.004%±0.004%), but not in the ZIKV-infected (0.009%±0.018%) or ACAM2000-vaccinated (0.005%±0.005%) samples (Fig 5C). These findings indicated to us that our diagnostic classifiers would likely work for independent cohorts of mice.

**Table 4:** TCRβ sequencing summary of independent mouse cohorts.

Treatment Group	# Samples	# Unique Clonotypes	Total TCR Rearrangements	Source Material
Naïve	n = 51	8.66x10^5^	1.89x10^6^	Manuscript & *Wolf et. al.[34]*
Zika	n = 14	5.43x10^4^	9.00x10^4^	Manuscript
Dengue-2	n = 5	3.00x10^4^	4.30x10^4^	Manuscript
ACAM2000	n = 29	3.92x10^5^	7.00x10^5^	*Wolf et. al. [34]*

Consolidated data referencing the total number of mice, unique TCRβ sequences (clonotypes), and total number of rearranged TCRβ genes sequenced from Naïve, Zika- or Dengue-2-infected, or ACAM2000 vaccinated mice in the independently tested cohort(s).

**Fig 6:**
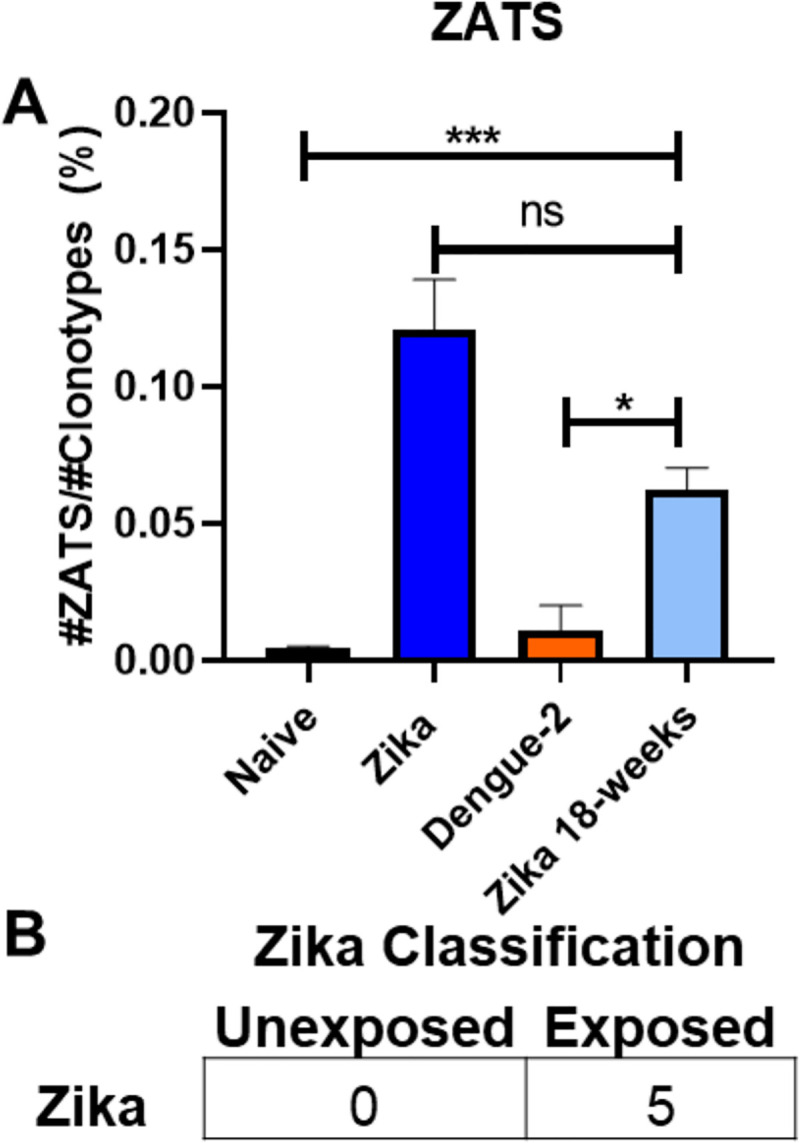
Durability of ZIKV diagnostic classifier. **(A)** The ZATS ratio was calculated for TCR sequenced peripheral blood lymphocytes of mice infected with ZIKV 18 weeks after exposure. Compared to 18 days post infection, there were no statistical differences in the ZATS ratio of the 18-week post infected mice. Statistical significance was determined by an unpaired t test. (**B**) The TCR sequences of 18-week post ZIKV exposure mice were run through the ZIKV diagnostic classifier and were correctly diagnosed as ZIKV exposed.

Finally, TCR sequences from these independent cohorts of mice were tested against the ZIKV and DENV2 diagnostic classifiers as the diagnostic assay to determine exposure to DENV2 or ZIKV simultaneously. From all samples tested, 100% of naïve (51 of 51) and ACAM2000-vaccinated mice (29 of 29) tested negative for both ZIKV and DENV2 (Fig 5D). Within this independent data set, 97% of the ZIKV infected mice were correctly classified as ZIKV infected, with only a single ZIKV infected mouse being categorized as naïve to both DENV2 and ZIKV. The durability of this diagnosis was also evaluated with a small cohort (5 of 5) of ZIKV infected mice, which were all correctly classified as ZIKV exposed out to 18-weeks post-infection (Fig 6A and 6B). 100% of the DENV2 infected mice (5 of 5) were correctly categorized as DENV2 infected, with 1 of the 5 samples also testing positive for ZIKV. Collectively, these data demonstrate that T cells with distinct TCRβ sequences expand and are enriched following ZIKV or DENV2 infection in mice, and that those distinct sequences can be used as a means of diagnostic discrimination between the two viruses in a highly sensitive assay. Moreover, this diagnostic classifier system is reproducible between multiple cohorts of mice and is durable enough to still be accurate 18 months after initial exposure.

## Discussion

Due to serological cross-reactivity, diagnostic differentiation between different flavivirus infections is a well-known challenge [2–7]. This fact is particularly troubling in the case of pregnant women with potential ZIKV exposure, due to the potential for lifelong debilitating consequences of infection to the fetus [16,47]. We believe that flavivirus specific T cell immune responses can be used as a reliable biomarker for a rapid flavivirus diagnostic test overcoming the current problems with the serological cross-reactivity.While T cells capable of *functionally* responding to heterologous flavivirus altered peptide ligands have been reported in humans [9], non-human primates [7,48], and mice [8], we demonstrate through TCRβ sequencing, discrete virus-specific TCR sequences can be used to computationally distinguish ZIKV infection from other flaviviruses, such as DENV2. This is an important proof-of-concept study using mice of known MHC-haplotype and flavivirus exposure status, which is a critical step before evaluating its potential use in complex human populations. This study builds on the groundbreaking studies already establishing T cells and their receptors as stable biomarkers, serving as a record of past pathogen exposures [27,29,32,33].

Within the study, we were able to computationally derive two sequencing libraries of public TCRs from the TCRβ sequences of T cells from ZIKV or DENV2 infected HLA-A2 transgenic mice. We showed that the TCRβ sequences that were enriched in the peripheral blood of ZIKV infected mice, termed “ZATS” were distinct from those enriched in the peripheral blood of DENV2 infected mice, termed “DATS.” We were able to link these high-throughput sequencing and bioinformatics approaches to functional immune assays in an integrative approach to show that these ZATS were particularly enriched in a population of sorted T cells responding to established ZIKV epitopes [35,36], further validating our libraries. When we evaluated our diagnostic classifiers against a cohort of DENV2 or ZIKV infected mice that were not part of our training data set, we were able to computationally identify these public TCRs, and correctly differentiate between ZIKV and DENV2 infection with 97% accuracy.

Categorizing antigen exposures by TCR-based sequencing has a number of strengths as a diagnostic approach. As an arm of the adaptive immune system, following infection and pathogen clearance, a subset of antigen-specific T cells will be retained at a higher frequency in immunological memory [27,28]. This naturally occurring phenomenon can therefore be equated to a data repository, cataloging an individual’s infection history. In using this technique, we and others have exploited this phenomenon for the purposes of accurate diagnosis of pathogen exposures [27,29,32,33]. The assay itself does not rely on T cell functionality as a readout; only the presence of the cell in peripheral blood. In this study we demonstrated that while an ELISA-based assay was unable to discriminate between DENV and ZIKV exposure, our TCR-based diagnostic approach was able to distinguish with substantial accuracy. While B cell receptor (BCR) sequencing could potentially be used to distinguish between primary DENV infection and ZIKV infection, its application in more complex co-infection scenarios would likely prove to be more challenging. This could potentially confound the diagnostic assay as affinity maturation of the BCR could occur following multiple flavivirus infections. In contrast, the TCR sequence is fixed and does not undergo affinity maturation following multiple exposures.

The geographic regions in which this technology would be most valuable are areas endemic for multiple flaviviruses and the target population would likely be individuals who have been exposed to multiple flaviviruses. So while we do acknowledge that due to the diversity HLA-types in humans, which may add addition layers of complexity in identifying public TCRs relative to the single haplotypes we used in our proof of concept study, we believe that our TCR sequencing approach can be used as an effective diagnostic. While relative frequencies of each virus-specific TCR may be altered with repeated flavivirus exposures, as they would be expected to be with exposure to any pathogen, the identifying sequence should remain present within the T cell repertoire as T cells do not undergo affinity maturation. Additionally despite the increased HLA-type diversity, studies in human cytomegalovirus infection have demonstrated that through sampling both a larger pool of the human population as well as a larger portion of the circulating T cells, an accurate diagnosis of cytomegalovirus infection can be made [27]. For this reason, we believe that accurate diagnosis in a complex immunological setting could be possible with more refined bioinformatics approaches and classifiers, which we are currently developing.

In conclusion, we have demonstrated that TCR sequencing to generate classification algorithms based on public virus-specific TCRs can be employed as an accurate diagnostic assay capable of discriminating between two closely related viruses (DENV and ZIKV) in this proof-of-principle murine study. While its initial testing is a critical step in the deployment of this technology as a laboratory diagnostic assay, further development is underway to design more complicated algorithms to address the real-world scenarios such as flavivirus co-infection and repeated exposures which currently confound serologically-based diagnostics. A diagnostic tool which can accurately distinguish ZIKV from related flaviviruses would inform surveillance and public health both within endemic regions and unaffected areas where the virus might spread.

## Supporting information

S1 Fig Human antibody binding specificity examined by capture ELISA using envelope protein. All human serum samples were collected from North American travelers six to nine months after identification as viremic by qRT-PCR. Red arrows indicate serum from ZIKV infected cross-reacts with all three flavivirus capture ELISAs shown.
(TIF)

S2 Fig Gating strategy for sorting of ZIKV-specific T cells. Following peptide stimulation in the presence of brefeldin A and staining (see materials and methods), ZIKV specific CD8+ or CD4+ T cells were sorted separately using the following gating strategy: Lymphocytes➔ CD19-➔ CD4+/CD8- or CD4-/CD8+➔ IFNγ+. Approximately 1% of the CD4+ T cells and 5% of the CD8+ T cells of ZIKV infected mice were sorted and sequenced.
(TIF)
