# Supplementary figures and images for: Diagnostic differentiation of Zika and dengue virus exposure by analyzing T cell receptor sequences from peripheral blood of infected HLA-A2 transgenic mice
Source: PLoS Negl Trop Dis. 2020 Dec 3;14(12):e0008896. doi: 10.1371/journal.pntd.0008896 (PMC7738164; doi:10.1371/journal.pntd.0008896)

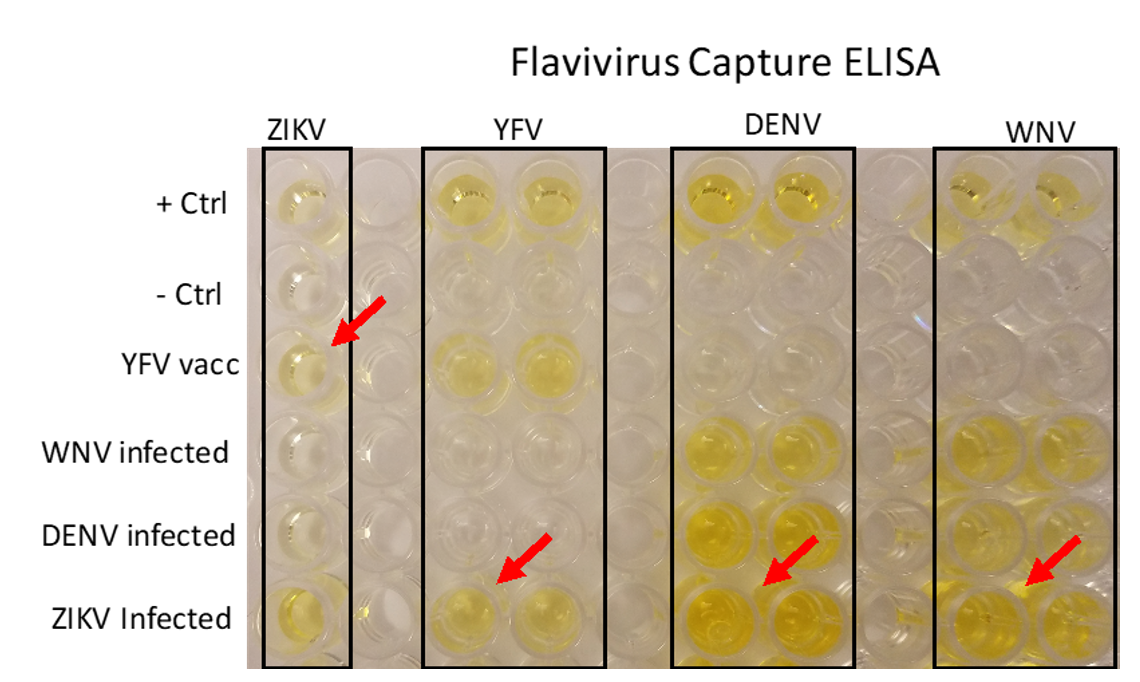

Supplement: S1 Fig — All human serum samples were collected from North American travelers six to nine months after identification as viremic by qRT-PCR. Red arrows indicate serum from ZIKV infected cross-reacts with all three flavivirus capture ELISAs shown. (TIF) [file pntd.0008896.s001.tif]

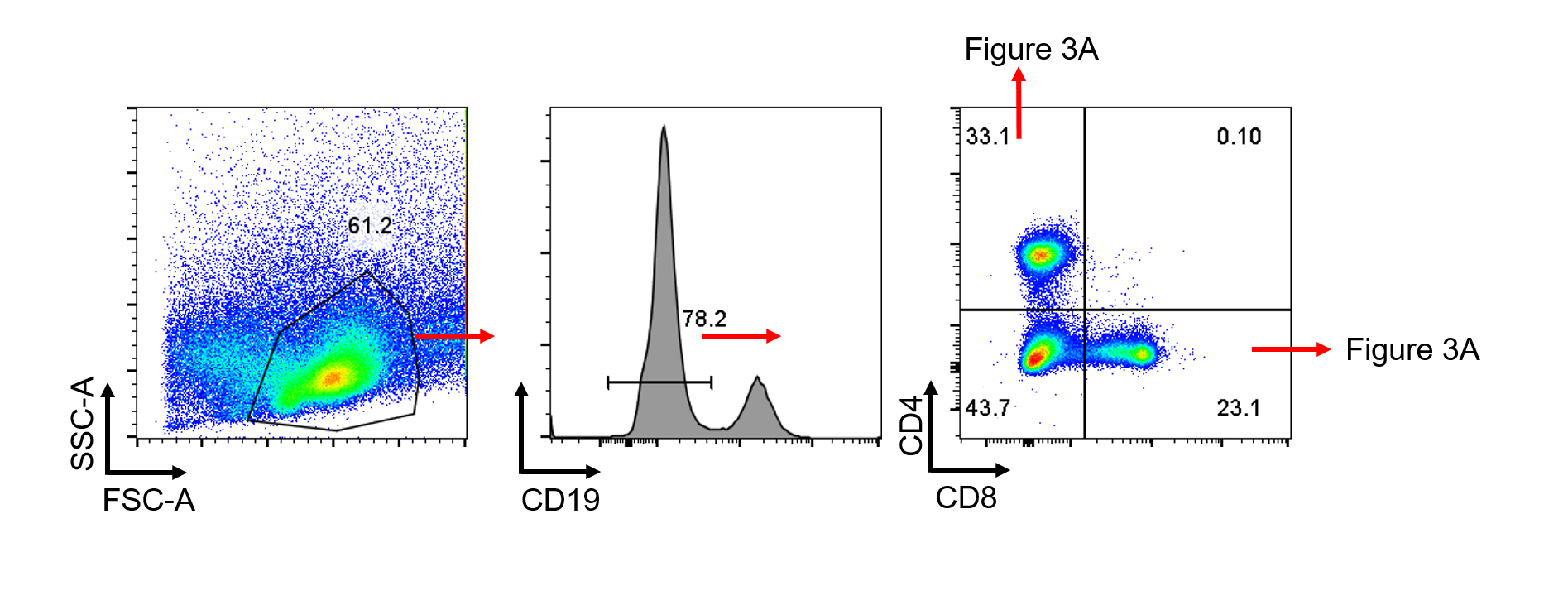

Supplement: S2 Fig — Following peptide stimulation in the presence of brefeldin A and staining (see materials and methods), ZIKV specific CD8+ or CD4+ T cells were sorted separately using the following gating strategy: Lymphocytes➔ CD19-➔ CD4+/CD8- or CD4-/CD8+➔ IFNγ+. Approximately 1% of the CD4+ T cells and 5% of the CD8+ T cells of ZIKV infected mice were sorted and sequenced. (TIF) [file pntd.0008896.s002.tif]
